# Modified Yuejuwan Inhibited Cholesterol Accumulation and Inflammation in THP-1 Macrophage-Derived Foam Cells by Inhibiting the Activity of the TRIM37/TRAF2/NF-*κ*B Pathway

**DOI:** 10.1155/2022/6400517

**Published:** 2022-03-10

**Authors:** Mingtai Gui, Lei Yao, Bo Lu, Jianhua Li, Jing Wang, Xunjie Zhou, Deyu Fu

**Affiliations:** Department of Cardiology, Yueyang Hospital of Integrated Traditional Chinese and Western Medicine, Shanghai University of Traditional Chinese Medicine, Shanghai, China

## Abstract

**Background:**

This study aimed to explore the function of modified Yuejuwan (MYJ) on THP-1 macrophage-derived foam cells.

**Methods:**

First, human THP-cells were obtained, and then, grouping was made to the following: control group, foaming group, foaming group +0.2 mg/mL Jiawei Yueju pill, foaming group +0.5 mg/mL Jiawei Yueju pill, and foaming group +1 mg/mL Jiawei Yueju pill. An Oil Red O staining assay was used to examine the uptake of oxidatively modified low-density lipoprotein (oxLDL). The secretion of interleukin (IL)-1*β* and tumor necrosis factor (TNF)-*α* were determined using an enzyme-linked immunosorbent assay (ELISA). Real-time quantitative PCR (qRT-PCR) and Western blot were used to quantify genes and proteins expression levels.

**Results:**

Our results indicated that MYJ inhibited the accumulation of total cholesterol (TC), free cholesterol (FC), and cholesteryl ester (CE) in foam cells. Moreover, the secretion of IL-1*β* and TNF-*α* also downregulated in foam cells after treatment of MYJ. Furthermore, we found that tripartite motif-containing 37 (TRIM37) was significantly upregulated in foam cells. Knockdown of TRIM37 promoted cholesterol efflux and presented an anti-inflammation effect in foam cells. Furthermore, TRIM37 positively mediated the translocation of NF-*κ*B to nuclear. It negatively regulated its ubiquitination in foam cells after interacting with TRAF2. Importantly, MYJ profoundly suppressed the function of TRIM37 in foam cells and functioned as a TRIM37 inhibitor.

**Conclusions:**

This study demonstrated that MYJ might alleviate oxLDL-induced foam cell formation by inhibiting the TRIM37/TRAF2/NF-*κ*B pathway activity. MYJ was a potential agent in preventing atherosclerosis and indicated its potential signaling pathway in foam cells.

## 1. Background

Atherosclerosis is a chronic inflammatory disease of the arterial wall, which is the most common underlying pathology of cardiovascular diseases (CVDs). CVDs are the most common cause of death worldwide, killing 17.5 million people, representing more than 30% of all global deaths [1, 2]. It is characterized by complex atherosclerotic plaques [3]. The process is deeply influenced by endothelial abnormal vascular inflammation, the proliferation of vascular smooth muscle cells, thrombus formation, and conversion of macrophages into foam cells [4]. Moreover, foam cells had been confirmed as the major culprit in atherosclerosis [5]. Targeting the formation of foam cells provides novel insight for fighting atherosclerosis and CVD [6].

Tripartite motif-containing 37 (TRIM37), containing RING finger region, is located in the 17q23 chromosomal region [7]. As an E3 ubiquitin ligase, TRIM37 is instrumental in regulating the inflammatory cascade [8]. Moreover, TRIM37 has a TNF-receptor associated factor (TRAF) sequence and enhances K63-linked ubiquitination of TRAF2 [9]. Furthermore, a previous report demonstrated that TRIM21 influences atherosclerosis through regulating Th17 [10]. Nuclear factor-*k*B (NF-*κ*B), a downstream factor of TRAF2, is reported as a proatherogenic factor [11], and activated NF-*κ*B is closely associated with foam cell formation and vascular inflammation [12, 13]. Moreover, it confirmed that NF-*κ*B inhibitors suppress the formation of foam cells and atherosclerotic plaque accumulation [14]. Therefore, inhibiting NF-*κ*B activity provided novel insight into the treatment for atherosclerosis.

Yuejuwan (YJ), a traditional Chinese medicine (TCM), is widely used for digestive dysfunction and depressive illness [15]. The newer version of the YJ, now being called modified Yuejuwan (MYJ), contributes to the formation of hepatic macrovesicular steatosis and reduces inflammatory infiltration in a nonalcoholic fatty liver disease rat model [16]. MYJ presented a clinical curative effect on removing dampness to reduce phlegm, invigorating the blood circulation, and eliminating stasis [17, 18]. However, the underlying effect of MYJ on foam cells is still unclear.

The target of the present study is to explore the effect of MYJ on human foam cells. Moreover, we also examined the role of TRIM37 in foam cells in the presence of MYJ. Our finding not only gained a deep insight into the biological effect of MYJ but also indicated its potential molecule pathway in human foam cells.

## 2. Methods

### 2.1. Study Design

A total of 207 g Jiawei Yueju was purchased and put in a special decoction pot, decocted on high heat until the water was boiled, fried slowly on low heat for about 45 minutes, and filter out 500 ml of liquid medicine. The medicinal solution was divided into a −80°C refrigerator and stored overnight and then put into a freeze-dried agent for freeze-drying. After 72 h, a powdered drug freeze-dried powder was obtained. Then, grouping was made as follows: control group, foaming group, foaming group +0.2 mg/mL Jiawei Yueju pill, foaming group +0.5 mg/mL Jiawei Yueju pill, and foaming group +1 mg/mL Jiawei Yueju pill. The basis for concentration selection is unknown.

### 2.2. Cell Culture

Human THP-1 cells were obtained from the cell bank of Shanghai Biology Institute (Shanghai, China). Cells were seeded in DMEM medium (Trueline, USA) containing FBS (10%, 16000–044, Gibco, USA) and penicillin-streptomycin solution (1%, P1400-100, Solarbio, China). All cells were incubated with the condition of 5% CO_2_ at 37°C. Then, THP-1 cells were primed with phorbol-12-myristate-13-acetate (PMA, 100 nM; P1585, Sigma, USA) for 24 h at 37°C to differentiate into macrophages and followed incubation with oxidatively modified low-density lipoprotein (oxLDL, 50 mg/L; H7950, Solarbio, China) for 48 h to transform these macrophages into foam cells [19–21].

### 2.3. Oil Red O Staining

Foam cells were fixed with paraformaldehyde solution for 30 min at room temperature after being washed twice with phosphate buffer solution (PBS). After that, cells were stained with Oil Red O for half an hour. The image of stained cells was collected using microscopy (XDS-600C, Caikon, Shanghai, China) on 200X.

### 2.4. RNA Extraction and Real-Time Quantitative PCR (qRT-PCR) Analysis

Total RNA was extracted using the TRIzol reagent kit (1596–026, Invitrogen, USA) and then converted into cDNA according to the instruction of the manufacture (#K1622, Fermentas, Canada). The experiment was established on real-time detection (ABI-7300, ABI, USA) using SYBR Green master mix (#K0223, Thermo, USA). Relative gene expression determination was counted according to the 2^−ΔΔCt^ method using *β*-actin as an endogenous reference. The primer sequences used in this study are given in Table 1.

### 2.5. Overexpression and Knockdown of TRIM37 in Foam Cells

For overexpression of TRIM37, the entire length of TRIM37 cDNA was inserted into the lentiviral vector (pLVX-Puro; Catalog No. 632164; Clontech Laboratories Inc., USA). Then, the recombinant vector (pLVX-Puro-oeTRIM37) was transfected into foam cells. Meanwhile, the mock vector functioned as the corresponding negative control (oeNC), and three short interfering RNAs (siRNA) that target different regions of human gene TRIM37 (NM_001005207.4) were synthesized and subsequently transfected into foam cells (siTRIM37-1, siTRIM37-2, and siTRIM37-3). A nonspecific scramble siRNA acted as the negative control (siNC). TRIM11siRNAs were provided as follows: siTRIM37-1 (1799–1817): GGAGAAGATTCAGAATGAA; siTRIM37-2 (2119–2137): CCAGTAGTTTACTAGACAT; siTRIM37-3 (2730–2748): GCCTTGATACATGGCAGTA.

### 2.6. Western Blotting

Total protein was isolated using RIPA lysis buffer (JRDUN, Shanghai, China). 25 *μ*g protein of each sample was fractionated and transferred onto PVDF nitrocellulose membrane (HATF00010, Millipore, USA) for 12 h. The transfer method was semidry at 25 V on 100 mA current, lasted for 30 minutes, and the pore size of the PVDF membrane was 0.22 *μ*m. Then, the membranes were probed with the primary antibodies overnight at 4°C, followed by the appropriate HRP-conjugated goat anti-rabbit IgG (A0208, Beyotime, China). Protein signals were analyzed using a chemiluminescence system. The antibodies details were provided as follows: TRIM37 (Ab95997, Abcam, UK, 1 : 1000), TRAF2 (Ab60169, Abcam, UK, 1 : 1500), SR-B1(Ab52629, Abcam, UK, 1 : 1000), ABCA1 (Ab18180, Abcam, UK, 1 : 1000), ABCG1 (Ab52617, Abcam, UK, 1 : 2000), NF-KB (#8242, CST, USA, 1 : 1000), H3 (#4499, CST, USA, 1 : 1000), and *β*-actin (#4970, CST, USA, 1 : 1000).

### 2.7. Enzyme-Linked Immunosorbent Assay (ELISA)

According to the manufacture protocol, initially, the protein cells were extracted from the incubator, and the culture solution was aspirated. Then, washed twice with precooled 1 × PBS, aspirated the PBS, added a lysis buffer containing protease and phosphatase inhibitors, and fully lysed at 4°C. The cells were then scraped into a 1.5 ml EP tube, heated at 95°C for 10 minutes, and centrifuged at 12000 g for 10 minutes. The supernatant was taken, and the protein was quantified and stored in a refrigerator at −80°C for WB detection. The secretion of IL-1*β* and TNF-*α* in foam cells was determined by ELISA kits (IL-1*β*: GS-E10083, X-Y Biotechnology, China; TNF-*α*: XY-E10110, X-Y Biotechnology, China).

### 2.8. Measurement of Lipid Levels

The free cholesterol (FC) content detection kit (cat#BC1895; specification: 100 T/96 S; Solarbio, China) and total cholesterol (T-CHO) test kit (cat#A111-1; COD-PAP method single reagent; Njjcbio, China) were purchased. According to the manufacture protocol, the levels of total cholesterol (TC) and FC were quantified. The difference value between TC and FC was counted as the content of cholesteryl ester (CE).

### 2.9. Coimmunoprecipitation (Co-IP)

In brief, cell lysates were isolated after transfection or stimulation with appropriate ligands. Then, all samples were incubated by TRIM37, TRAF2, SR-B1, ABCA1, ABCG1, NF-KB, H3, and *β*-actin antibodies plus protein A/G beads (SantaCruz Biotechnology, USA) overnight before they were separated by SDS-PAGE.

### 2.10. Ubiquitination Assay

Foam cells transfected with siNC or siTRIM37 were lysed by sonication in 1% SDS-containing radioimmunoprecipitation assay (RIPA) buffer on ice. Then, lysates were treated by protein A/G plus agarose (sc-2003, Santa Cruz Biotechnology, USA) for 1 h. After that, each sample was incubated with IgG (sc-2027, Santa Cruz Biotechnology, USA) overnight at 4°C. Then, the nuclear pellet was gathered by centrifugation at 3000 rpm for 5 min at 4°C and subsequently washed by protein A/G Plus-Agarose beads four times. The purified proteins were run on 4–20% gradient SDS-PAGE. Anti-TRAF2 antibody (Ab60169, Abcam, UK) and antiubiquitin antibody (ab7780, Abcam, UK) were used for immunoblotting.

### 2.11. Data and Statistical Analysis

All values are expressed as means ± S.E.M and analyzed with GraphPad Prism software V7.0 (California, USA). The results were assessed by analysis of variance (ANOVA) with LSD. Each analysis was performed in triplicate for all the procedures. *P* values <0.05 were considered statistically significant.

## 3. Results

### 3.1. OxLDL Induced the Formation of Human THP-1 Macrophage Foam Cells

**Figure 1:**
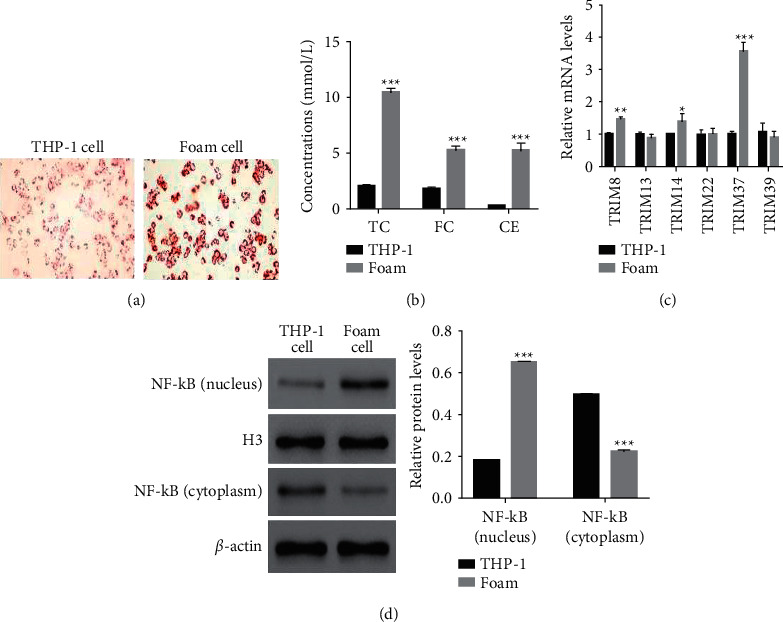
OxLDL induced the formation of human THP-1 macrophage foam cells. (a) Oil Red O staining indicating that oxLDL uptake upregulated in foam cells. Magnification: 200x. (b). The contents of TC, FC, and CE increased in foam cells. ^*∗∗∗*^*P* < 0.001 vs. THP-1. (c) qRT-PCR used to examine the relative mRNA levels of TRIM8, TRIM13, TRIM14, TRIM22, TRIM37, and TRIM39 in THP-1 and foam cells, respectively. ^*∗*^*P* < 0.05 vs. THP-1, ^*∗∗*^*p* < 0.01 vs. THP-1, ^*∗∗∗*^*p* < 0.001 vs. THP-1. (d) The translocation of NF-*κ*B promoted in foam cells. ^*∗∗∗*^*P* < 0.001 vs. THP-1.

Oil Red O staining assay was used to determine oxLDL uptake in foam cells and revealed that oxLDL uptake was much higher in foam cells than in THP-1 cells (Figure 1(a)). In addition, TC, FC, and CE were quantified and found that accumulation of TC, FC, and CE significantly upregulated in foam cells than that of THP-1 cells (Figure 1(b)). The relative mRNA levels of TRIM8, TRIM14, and TRIM37 were upregulated in foam cells, especially for TRIM37 (Figure 1(c)). The translocation of NF-*κ*B to nuclear also increased in foam cells (Figure 1(d)).

### 3.2. MYJ Inhibited the Accumulation of Cholesterol in THP-1 Macrophage-Derived Foam Cells

**Figure 2:**
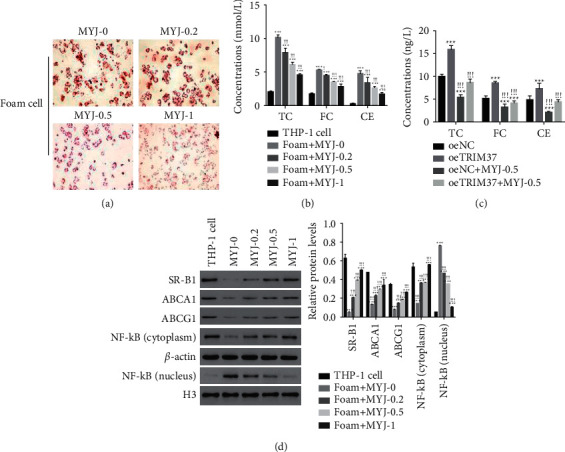
MYJ increased cholesterol efflux in THP-1 macrophage-derived foam cells. (a) MYJ treatment reducing oxLDL uptake in foam cells. Magnification: 200x. (b) MYJ treatment inhibiting the accumulation of TC, FC, and CE in foam cells. ^*∗∗∗*^*P* < 0.001 vs. THP-1, ^!^*p* < 0.05 vs. foam + MYJ-0, ^!!!^*p* < 0.001 vs. foam + MYJ-0. (c) The contents of IL-1*β* and TNF-*α* decreased in foam cells in the presence of MYJ. ^*∗∗∗*^*P* < 0.001 vs. THP-1; ^!!^*p* < 0.01 vs. foam + MYJ-0, ^!!!^*p* < 0.001 vs. foam + MYJ-0. (d) Western blot used to examine the protein contents of SR-B1, ABCA1, ABCG1, NF-*κ*B (cytoplasm), and NF-*κ*B (nucleus) in cells as indicated above. ^*∗∗∗*^*P* < 0.001 vs. THP-1; ^!!!^*p* < 0.001 vs. foam + MYJ-0.

Foam cells were cultured with MYJ at different concentrations, including 0, 0.2, 0.5, and 1 mg/mL, to assess the function of MYJ on foam cells. As shown in Figure 2(a), the MYJ treatment suppressed the uptake of oxLDL in foam cells as the concentration rising. Moreover, the production of TC, FC, and CE downregulated in foam cells in the presence of MYJ (Figure 2(b)). Besides, IL-1*β* and TNF-*α* are important proinflammatory cytokines [22]. In this analysis, MYJ treatment decreased the expression of IL-1*β* and TNF-*α* in foam cells (Figure 2(c)).

Moreover, scavenger receptor-B1 (SR-B1), ATP-binding cassette transporter A1 (ABCA1), and ATP-binding cassette transporter G1 (ABCG1) prevent the accumulation of cholesterol in cells by promoting cholesterol efflux to lipid-poor apolipoprotein A-I [23]. Our results suggested that the protein contents of SR-B1, ABCA1, and ABCG1 profoundly decreased in foam cells than in THP-1 cells, whereas significantly recovered by MYJ treatment in a dose-dependent manner (Figure 2(d)). Taken together, these results suggested that MYJ might contribute to cholesterol efflux by positively regulating ABCA1, ABCG1, and SR-B1 in foam cells.

### 3.3. Overexpression and Knockdown of TRIM37 in Foam Cells

**Figure 3:**
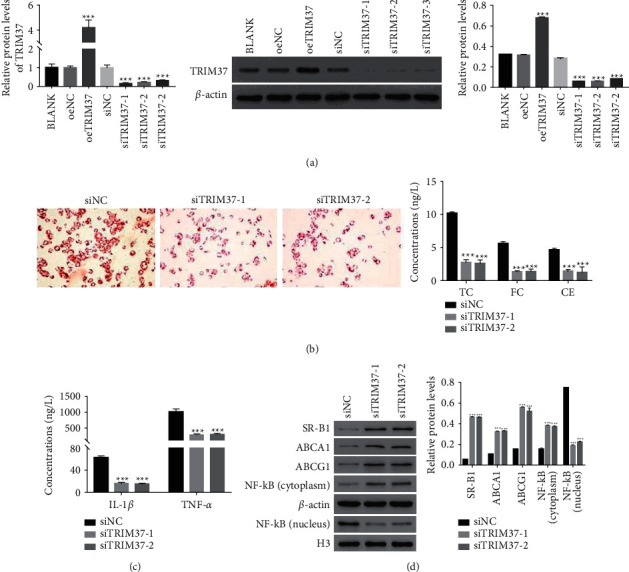
Overexpression and knockdown of TRIM37 and its silencing promoted cholesterol efflux in foam cells. (a) The relative mRNA levels and relative protein contents of TRIM37 in different transfected cells as indicated. ^*∗∗∗*^*P* < 0.001 vs. oeNC. (b) TRIM37 silencing inhibiting the uptake of oxLDL in foam cells. Magnification: 200x. TC, FC, and CE contents downregulated in siTRIM37-1 or siTRIM37-2 transfected cells. ^*∗∗∗*^*P* < 0.001 vs. siNC. (c) Knockdown of TRIM37 inhibiting the secretion of IL-1*β* and TNF-*α* in foam cells. ^*∗∗∗*^*P* < 0.001 vs. siNC. (d) Western blot used to quantify the protein content of SR-B1, ABCA1, ABCG1, NF-*κ*B (cytoplasm), and NF-*κ*B (nucleus) in cells as indicated above. ^*∗∗∗*^*P* < 0.001 vs. siNC.

To address TRIM37 function in foam cells, TRIM37 induced overexpression and knockdown in foam cells. The relative mRNA and protein levels of TRIM37 remarkably increased in oeTRIM37 transfected cells. As shown in Figure 3(a), all TRIM37 siRNAs significantly disrupted the endogenous expression of TRIM37 in foam cells, especially for siTRIM37-1 and siTRIM37-2, which to be further investigated in a future study with extended experimental plans.

### 3.4. TRIM37 Silencing Promoted Cholesterol Efflux in Foam Cells

Then, we examined the uptake of oxLDL in siTRIM37 transfected cells. The accumulation of oxLDL in siTRIM37 transfected cells decreased greatly. Both siTRIM37-1 and siTRIM37-2 have deeply suppressed the production of TC, FC, and CE in foam cells (Figure 3(b)). Importantly, our results revealed that the expression of IL-1*β* and TNF-*α* deeply decreased in siTRIM37-1 or siTRIM37-2 transfected cells (Figure 3(c)). More importantly, TRIM37 silencing significantly suppressed the translation of SR-B1, ABCA1, and ABCG1 in foam cells. These findings suggested that siTRIM37 might promote cholesterol efflux by inhibiting ABCA1, ABCG1, and SR-B1 in foam cells (Figure 3(d)).

### 3.5. TRIM37 Interacted with TRAF2 and Negatively Mediated Its Ubiquitination in Foam Cells

**Figure 4:**
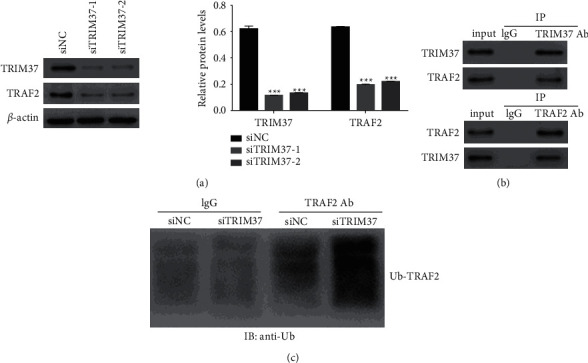
TRIM37 interacted with TRAF2 and negatively mediated its ubiquitination in foam cells. (a) The protein content of TRAF2 suppressed in siTRIM37-transfected cells. ^*∗∗∗*^*P* < 0.001 vs. siNC. (b) TRIM37 interacting with TRAF2 in foam cells. (c) Knockdown ofTRIM37 enhancing the ubiquitination of TRAF2 in foam cells.

Next, we examined the protein content of TRAF2 in siTRIM37-1 or siTRIM37-2 transfected cells. As shown in Figure 4(a), the protein content of TRAF2 significantly downregulated in siTRIM37-1 or siTRIM37-2 transfected cells. Moreover, results from the Co-IP assay indicated that TRIM37 interacted with TRAF2 in foam cells (Figure 4(b)). Notably, the knockdown of TRIM37 enhanced the ubiquitination of TRAF2 in foam cells (Figure 4(c)). Therefore, TRIM37 silencing might suppress the translocation of TRAF2 via enhancing its ubiquitination in foam cells.

### 3.6. MYJ Inhibited the Function of TRIM37 in Foam Cells

**Figure 5:**
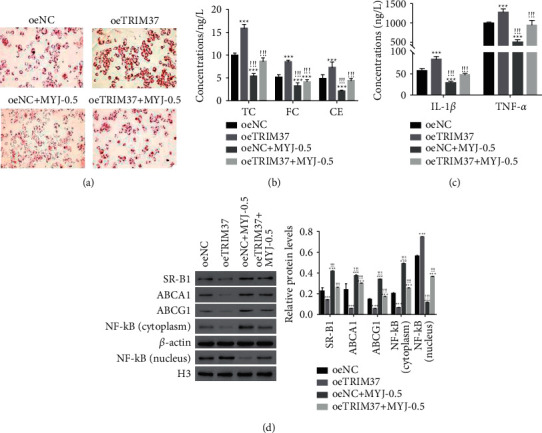
MYJ functioned as a TRIM37 inhibitor in foam cells. (a) MYJ treatment inhibiting the uptake of oxLDL in oeTRIM37-transfected cells. Magnification: 200x. (b) The accumulations of TC, FC, and CE downregulated in oeTRIM37-transfected cells in the presence of MYJ. ^*∗∗∗*^*P* < 0.001 vs. oeNC; ^!!!^*p* < 0.001 vs. oeTRIM37. (c) The secretion of IL-1*β* and TNF-*α* inhibited in oeTRIM37-transfected cells with the treatment of MYJ. ^*∗∗∗*^*P* < 0.001 vs. oeNC; ^!!!^*p* < 0.001 vs. oeTRIM37. (d) Western blot used to examine the contents of SR-B1, ABCA1, ABCG1, NF-*κ*B (cytoplasm), and NF-*κ*B (nucleus) in cells as indicated above. ^*∗∗∗*^*P* < 0.001 vs. oeNC; ^!!!^*p* < 0.001 vs. oeTRIM37.

To further address the relationship between TRIM37 and MYJ, the oeTRIM37 transfected cells were cultured in the presence of MYJ (0.5 mg/mL). TRIM37 overexpression promoted the uptake of oxLDL in foam cells, but this function was suppressed with the treatment of MYJ (Figure 5(a)). Moreover, the contents of TC, FC, and CE were also deeply suppressed by MYJ in oeTRIM37 transfected cells (Figure 5(b)). Furthermore, our results revealed that TRIM37 overexpression promoted the expression of IL-1*β* and TNF-*α* in foam cells. However, this effect was also disrupted with the treatment of MYJ (Figure 5(c)). Meanwhile, TRIM37 overexpression promoted the translocation of NF-*κ*B to nuclear in foam cells (Figure 5(d)). Taken together, these results demonstrated that MYJ presented the effects as a TRIM37 inhibitor in foam cells.

## 4. Discussion

Modified Yuejuwan (MYJ), a traditional Chinese medicine (TCM) widely used for mood disorders, has shown a curative effect on reducing phlegm, stimulating blood circulation, and eliminating stasis [15, 17, 18]. The underlying effect of MYJ in atherosclerosis remains unknown. This study explored the biological effect of MYJ and revealed its potential molecular pathway in human foam cells. After treating with MYJ, the secretion of IL-1*β* and TNF-*α* was downregulated, whereas TRIM37 was upregulated in foam cells. MYJ is effective for relieving depression, regulating digestion, and improving the emotional state [24]. So far, MYJ was found to be effective in the glutamate-induced HT22 cell injury model and used in different diseases [25, 26].

Foam cells and macrophages with engorging cholesterol are the major causes of atherosclerosis [27]. Therefore, inhibiting the transform of macrophages into foam cells draws much attention in the treatment of atherosclerosis. Fullerene derivatives, vitamin E, and apolipoprotein A-I mimetic peptide D4F can ameliorate the formation of oxLDL-induced foam cells [14, 28, 29]. In this study, we investigated the function of MYJ in THP-1 macrophage-derived foam cells. The findings of this study elucidated the potential value of MYJ in the prevention of atherosclerosis.

TRIM37 is known for its RING domain, B-box motifs, a coiled-coil region, and a C-terminal part that includes the MATH domain. TRIM37 deficiency is plagued with numerous tumors. A wide variety of cancers are associated with overexpression of TRIM37 [30]. TRIM37 is also an essential determinant of mitotic vulnerability to PLK4 inhibition [31]. Nonetheless, TRIM37 participates in K63 polyubiquitination of TRAF2, which is a significant step in the activation of NF-*κ*B signaling [9].

NF-*κ*B activation is widely recognized as a pathological mechanism of the lipid metabolism and atherosclerosis [32]. On the other hand, TRAF2 is reported as an adaptor factor and mediates NF-*κ*B activation [33]. Moreover, TRIM37 ubiquitylates PEX5 at K464 to promote peroxisomal matrix protein import [34]. In this analysis, our results indicated that MYJ inhibited the translocation of NF-*κ*B to nuclear and suppressed the translation of TRAF2 in foam cells. In addition, TRIM37 was identified as upregulation in THP-1 macrophage-derived foam cells. Moreover, TRIM37 interacted with TRAF2 and negatively mediated its ubiquitination in foam cells. TRIM37 possibly serves different functions in regulating TRAF2 ubiquitination in different cells.

Importantly, we found that MYJ functioned as a TRIM37 suppressor in foam cells. Taken together, all these findings indicated that MYJ might alleviate oxLDL-induced foam cell formation by inhibiting the activity of the TRIM37/TRAF2//NF-*κ*B pathway.

## 5. Conclusions

In this study, we investigated the function of MYJ in THP-1 macrophage-derived foam cells and explored its potential signaling pathway. Our findings demonstrated that MYJ was a promising agent in the treatment of atheroma and indicated its potential signaling pathway in foam cells.

## Figures and Tables

**Table 1:** Sequences for the primers used in the research.

Name	Primer sequences (5′-3′)
TRIM8F	5′-CAGCCGTCCACCAAACACTAC-3′, R 5′-ACCTCTGCGTCCAGGAGATTC-3′
TRIM13F	5′-TGCCCAGTAGCCTCTAGTTC-3′, R 5′-GGCCAGGTGCTGTTATTCTC-3′
TRIM14F	5′-GGATTTGTGTCTCCGTTCTG-3′, R 5′-TCTGTCTGCCTGGTATTCTG-3′
TRIM22F	5′-TCCATAGCAAAGCATCATAG-3′, R 5′-GACAATGTGAAGAGTCATAG-3′
TRIM37F	5′-TGGACTTACTCGCAAATG-3′, R 5′-ATCTGGTGGTGACAAATC-3′
TRIM39F	5′-GATCACTCGCTGCAAGTCC-3′, R 5′-CACCTGCTGCTCTTCATCC-3′
ACTBF	5′-GATGACCCAGATCATGTTTGAG-3′, R 5′-TAATGTCACGCACGATTTCC-3′

## Data Availability

The datasets used and/or analyzed during the current study are available from the corresponding author upon request.

## Abbreviations

MYJ: Modified Yuejuwan
oxLDL: Oxidatively modified low-density lipoprotein
IL: Interleukin
TNF: Tumor necrosis factor
ELISA: Enzyme-linked immunosorbent assay
qRT-PCR: Quantitative PCR
TRIM37: Tripartite motif-containing 37
CVDs: Cardiovascular diseases
TRAF: TNF-receptor associated factor
NF-*κ*B: Nuclear factor-*k*B
TCM: Traditional Chinese medicine
PBS: Phosphate buffer solution
siRNA: Short interfering RNAs
oeNC: Corresponding negative control
FC: Free cholesterol
T-CHO: Total cholesterol
CE: Cholesteryl ester
Co-IP: Coimmunoprecipitation
RIPA: Radioimmunoprecipitation assay.
